# Testing the Stability of Drug Resistance on Cryopreserved, Gene-Engineered Human Induced Pluripotent Stem Cells

**DOI:** 10.3390/ph14090919

**Published:** 2021-09-11

**Authors:** Dilaware Khan, Ann-Christin Nickel, Sebastian Jeising, Constanze Uhlmann, Sajjad Muhammad, Daniel Hänggi, Igor Fischer, Ulf Dietrich Kahlert

**Affiliations:** 1 Department of Neurosurgery, Medical Faculty, University Hospital Düsseldorf, Heinrich Heine University Düsseldorf, 40225 Düsseldorf, Germany; Dilaware.Khan@med.uni-duesseldorf.de (D.K.); Ann-Christin.Nickel@med.uni-duesseldorf.de (A.-C.N.); Sebastian.Jeising@med.uni-duesseldorf.de (S.J.); constanze.uhlmann@hhu.de (C.U.); Sajjad.Muhammad@med.uni-duesseldorf.de (S.M.); Daniel.Haenggi@med.uni-duesseldorf.de (D.H.); Igor.Fischer@med.uni-duesseldorf.de (I.F.); 2 Department of Molecular and Experimental Surgery, Clinic for General, Visceral, Vascular, and Transplant Surgery, Medical Faculty and University Hospital Magdeburg, 39108 Magdeburg, Germany

**Keywords:** stem cells, repeatability, cryopreservation, drug testing, MYC, TP53

## Abstract

Human induced pluripotent stem cells (hiPSCs) have emerged as a powerful tool for in vitro modelling of diseases with broad application in drug development or toxicology testing. These assays usually require large quantities of hiPSC, which can entail long-term storage via cryopreservation of the same cell charges. However, it is essential that cryopreservation does not oppose durable changes on the cells. In this project, we characterize one parameter of functionality of one that is well established in the field, in a different research context, an applied hiPSC line (iPS11), namely their resistance to a medium size library of chemo interventions (>160 drugs). We demonstrate that cells, before and after cryopreservation, do not change their relative overall drug response phenotypes, as defined by identification of the top 20 interventions causing dose-dependent reduction of cell growth. Importantly, also frozen cells that are exogenously enforced for stable overexpression of oncogenes myelocytomatosis (cMYC) or tumor protein 53 mutation (TP53R175H), respectively, are not changed in their relative top 20 drugs response compared to their non-frozen counterparts. Taken together, our results support iPSCs as a reliable in vitro platform for in vitro pharmacology, further raising hopes that this technology supports biomarker-associated drug development. Given the general debate on ethical and economic problems associated with the reproducibly crisis in biomedicine, our results may be of interest to a wider audience beyond stem cell research.

## 1. Introduction

In some instances, cancer research using classical cancer models derived from tumor specimens suffers from a limited rate of repeatability and reproducibility of results [1,2,3]. Human induced pluripotent stem cells (hiPSCs)—with their multifaceted in vitro and in vivo application possibilities—are considered one of the key technologies for future innovation of industrialized nations. One field of application of hiPSC, characterized with an increasing global annual market size, is the use of hiPSCs as in vitro disease models to test pharmacological interventions (www.researchandmarkets.com, accessed on 20 July 2021). Within this market, the use of cells with genetic alterations to generate analogous synthetic tumor cells to find anti-stem cell directed cancer therapies is emerging [4,5,6]. A central technology requirement to ensure long-term durability of the dissemination of hiPSC applications is the ability of such cells to survive freezing storage conditions meanwhile preserving their genetic and functional stability. Various prominent institutionally funded network activates, such as the European Bank for Induced Pluripotent Stem Cells [7], grant access to scalable, cost-efficient and consistent, high quality hiPSCs. Despite this enormous importance of hiPSC for future medicine, and although heavy bio-banking activities are underway, there is insufficiency of knowledge of cryopreservation procedures, one essential step in almost all in vitro applications/products, affect the functional repeatability of banked iPSCs. To our knowledge there is no paper available describing the testing of drug response profiles of oncogene-engineered hiPSCs upon cryopreservation. We believe our study is of importance for different members of the stakeholder chain in biomedical research. 

## 2. Results

The profile recordings revealed interpretable data, featuring a concentration-dependent effect on cell growth for most conditions. Therefore, our setup established a robotic-mediated semi-automated hiPSC in vitro screening technology platform suitable to record and to score the amplitude of concentration-dependent anti-growth effects of test substances.

**Figure 1:**
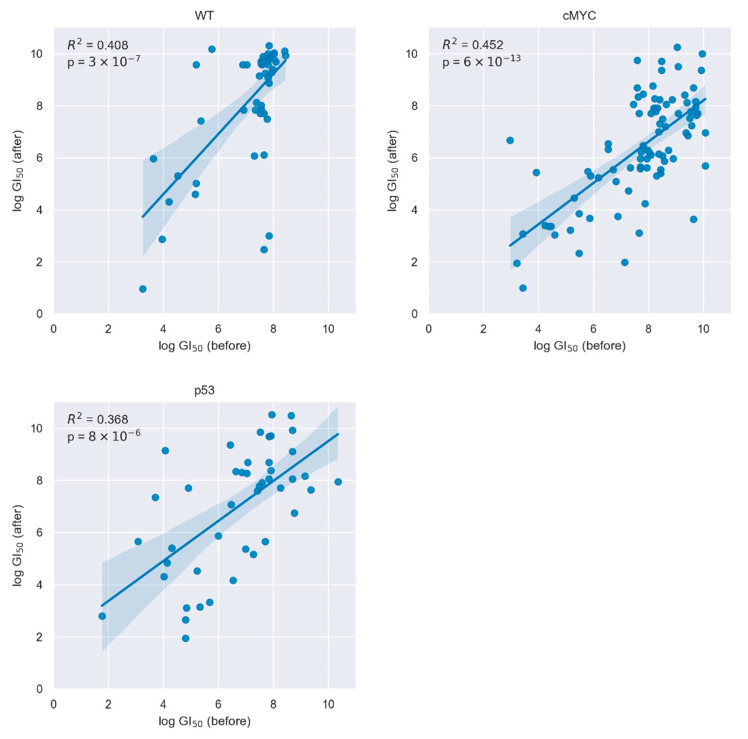
The relative drug response of the top 20 drugs stayed similar upon cryopreservation. Scatterplots showing the linear regression between GI50 log concentration of the 20 most responsive drugs prior to cryopreservation and the corresponding drug response after cryopreservation.

Next, for the described three different genetic conditions, we recorded the cell growth of cell pairs before and after freezing in response to exposure to >160 different drugs. Focusing on the 20 top performer drugs (defined by dose-dependent reduction of cells growth with minimal used drug concentration), we found that the cells maintained their relative drug resistance after thawing. Our statistical evaluation reveals that when analyzing the entire screening data there is a correlation of the drugs showing top effectivity in both time points (Figure 1). Separated for the three cell lines, Table 1, Table 2 and Table 3 list the names of the top performing drugs and their efficacy ranking before and after freezing (designated as “stable” drug). In the same tables, we name drugs that did not show up as top performers after freezing, scoring outside of the top 20 ranked interventions. In addition, inability to determine an unequivocal dose-dependent effect of the drug at the later time point could be a reason for a classification of a drug as “unstable”.

Analyzing the drug response profiles of cells per genetic condition in the acquired data from the entire library before and after freezing, our data showed that in all test models, we had identified an overall correlation of the top 20 performing drugs between the two time points. Moreover, we could identify in the IPS11-WT 13 from 20 drugs that react in a similar way, and 8 of those drugs were both in the top 20 prior and after freezing. For the disease modelling of hiPSCs-cMYC and -TP53, we observed similar results. Seventeen or nineteen drugs worked in cell lines prior and after freezing in iPS11-TP53 and iPS11-cMYC, respectively. Furthermore, in both models 60–70% of the working drugs were in the top 20 cell killing drugs.

## 3. Discussion

Many previous studies have devoted their work to testing reproducibility characteristics of hiPSCs-derived specialized cells upon cryo-storage, such as their survival percentage and differentiation purity [8,9,10,11,12]. Although others recently published suggestions on technical guidelines for optimal cryopreservation and transportation of stem cells in order to ensure high applicability of the cells in the context of cell therapy [13,14], dedicated studies benchmarking the reproducibly properties of hiPSCs—either genetically or cell biologically—are rarely available. Most of the studies on hiPSC and cryopreservation concentrate on defining optimal cryo-media and its supplements or on freezing instrumentation or handling procedures, as recently summarized by Horiguchi and Kino-oka [15], neglecting the testing of more complex functionality of genetic properties upon revitalization. To our knowledge, one relevant study recently originated from a major industry player in the life science market using three cell lines. The authors working for Lonza published their GMP protocol that enables the generation of long-term stabile, allowing repeatable differentiation procedures of hiPSCs [16]. In contrast to our results, another very recent report on drug resistance stability upon cryopreservation in hiPSC-derived differentiated cells (=cardio myocytes) showed that the response profile was changed in cells when thawed, possibly due to the observed alterations in transcriptome and the electro-mechanical functions of the cells [17]. 

Our results, showing a linear relationship between the top drugs before and after thawing and freezing, evaluating the total 167 different conditions, are in many aspects novel. Firstly, to our knowledge, this is one of the first reports on reproducible drug resistance capacity in cryopreserved iPSC per se. Secondly, drug screening assays with clinically approved drugs emerges as a powerful functional OMICs assay accompanying genomics, proteomics or metabolomics of cancer in vitro models to identify personalized therapy vulnerabilities or drug targets [18]. We present an independent dataset on FDA-cancer drug screening for a collection of isogenic hiPSC models, generating ground work for similar network fusion campaigns [19] devoted to stem cell-devoted questions. Lastly, in the context of iPS11 and c-MYC or TP53R175H, this is the hitherto first indication that the repeatability of the relatively strongest drug effects upon cryopreservation can also be maintained in hiPSCs forced to overexpress cancer genes—even when the genes were introduced in an untargeted fashion using an uncontrolled lentiviral-mediated procedure. This is particularly important as genetic manipulation of hiPSC, such as the stepwise introduction of an oncogenic signal in PSCs derived from healthy donors, is considered a promising way to develop synthetic cancer stem cells that may serve as a drug screening pipeline for B2B or B2C businesses. 

We are aware that future work on validating the herein-presented results, such as testing on multiple cell lines thereby increasing the ethic and gender diversity of the test matrix or increasing the number oncogenic mutations and combinations per cell model as well as comparing the repeatability of cells induced with different reprogramming methods, must prove the broader context of our results. Moreover, confirmative studies on oncogene-activated hiPSC-derived tissue-specific stem cells, such as oncogene-activated intestinal or neuronal stem cells, are needed to draw disease-specific annotations from the biomarker-attributed drug response profiles on hiPSC-based in vitro systems. We believe that, at this moment in time and in the context of oncology, therapeutically meaningful data requires still confirmative studies using patient-derived (cancer) cell models and animal models.. However, with the emergence of hiPSC-based organoids or assembloids as animal-free test matrixes, which are able to recapitulate more complex tissue organizations, our research is well in line with the global 3R movement. Moreover, institutionally funded network activities from leading science institutions or policy makers aim to enforce the establishment of reproducible stem cell procedures to increase the translation potential of products derived thereof [20,21]. We believe our project is in support of their underlying concepts and goals.

## 4. Material and Methods

### 4.1. Cell Growth and Cell Models

The cells were grown in a defined medium for human embryonic stem cells (mTeSR medium) (STEMCELL Technologies, Vancouver, BC, Canada) on Matrigel-coated (Corning, NY, USA) 6-well plates. The coated plates were kept at 4 °C and were used within two weeks. The medium was changed every day and the cells were passaged at 80–90% confluence. To passage the hiPSCs, cells were washed twice with 0.5 mM ethylenediamine tetraacetic acid (EDTA) in phosphate buffered saline (PBS), followed by incubation with 1 mL 0.5 mM EDTA at 37 °C for 5 min. The EDTA was removed and detached cells were collected using 1 mL of mTeSR medium. The cells were passaged in 1:8 to 1:12 depending on the confluence and were seeded on the already Matrigel-coated plates.

IPS11, an episomal-derived, foreskin fibroblast-originated hiPSC line, was purchased from Alstem Inc., Richmond, CA, USA, in the year 2018. All our cells continually pass our quality control system [22] to exclude contamination, accumulation of karyotype abnormalities or loose of pluripotency. Overexpression of cMYC and TP53R175H in iPS11 was achieved according to the procedure described recently [23]. In our work, ethics follow general bioethics guidelines related to biomedical research, such as the Declaration of Helsinki. We exclude dual use of our research. The use of the humaninduced pluripotent stem cells was recorded at the local government (Bezirksregierung Düsseldorf), especially in regard to their genetic modification. 

### 4.2. Cryopreservation Procedure

The cells were cryopreserved when they had reached a confluence of about 70–80%. Prior to cryopreservation, cells were washed with 1 mL 0.5 mM EDTA followed by incubation in 1 mL 0.5 mM for 5 min at 37 °C. The EDTA was aspirated and cells were detached using 1 mL of D10 NutriFreeze medium. The cells were then transferred into freezing tubes and placed in a freezing container (Thermo Fisher Scientific™, Waltham, MA, USA) and kept at −80 °C in a freezer overnight. The next day the frozen vials were transferred to the liquid nitrogen storage.

For thawing, the vials were removed from the liquid nitrogen tank and warmed at 37 °C using a water bath. Close to complete thawing, the cells were transferred into a centrifugation tube and 1 mL of cold mTeSR medium was added dropwise. The cells were centrifuged and washed under standard condition (300 g, 5 min), and the supernatant was removed and the cells were carefully re-suspended in mTeSR medium and plated into 6-well plates, reaching a final volume of 2 mL. The cells were passaged at least 3 times for expansion prior to usage in experiments or cryopreservation. Strong adherence to defined cryopreservation procedures and maintenance of temperature stability as controlled by a chronic temperature logging system, as well as validation of the genetic authentication of our cells, was achieved via applying our electronic lab notebook system [24] and lab quality control system [22]. 

### 4.3. In Vitro Pharmacology Testing

First, 384-well plates were coated with Matrigel (1:60) in mTeSR medium using our robot technology (Beckman Coulter Biomek^®^ FxP robotic workstation with attached micro-plate reader (Paradigm, now Molecular Devices, San Jose, CA, USA) [25]. After coating, the plates were shortly downcentrifuged and sealed using parafilm. Single cell suspension of the hIPSCs was prepared using StemPro Accutase Cell Dissociation Reagent (Thermo Fisher Scientific™, Waltham, MA, USA) containing 10 µM Rock inhibitor (Selleck Chemical Llc., Houston, TX, Houston). hiPSCs wt were used at passage 18 (7) and 19 (6), hiPSCs cMYC were used at passage 37 (4) and 38 (5), hiPSCs P53 were used at passage 44 (5) and 45 (6). In detail, the cells were washed twice with PBS followed by treatment with 1 mL accutase in the incubator with 5% CO_2_ at 37 °C for 4–5 min. The mTeSR medium was added to stop the reaction and the cells were centrifuged at 200 g for 5 min. The supernatant was removed and the cells were suspended in fresh mTeSR medium and counted using Trypan Blue (Thermo Fisher Scientific™, Waltham, MA, USA). For the screening, 2000 cells per well were applied in 40 µL mTeSR medium into a 384-well plate using a Biomek^®^ FxP robotic workstation. The next day, the cells were washed with PBS (Ca^++^ Mg^++^) (Thermo Fisher Scientific™, Waltham, MA, USA), and fresh mTeSR medium was added and 167 drugs in mTeSR with 5 concentrations ranging from 2 nM to 20 µM were applied to the cells. The exact listing of the composition of the drug library can be found elsewhere [25]. The cells were then incubated for 48 h, after which the readout of the cell survival was performed using the luminescence-based CellTiterGlo assay (Promega, Walldorf, Germany) according to manufacturer guidelines, except that we diluted the reaction agent 1:1 with PBS.

### 4.4. Statistical Evaluation of Repeatability of Drug Responses

Linear regression was used to model the relationship between growth inhibition 50% (GI50) for the fresh and cryopreserved cells, over all substances for which GI50 was reached and for which it could be numerically determined. All computations were performed in Python, Version 3.9.4. For statistical modelling, the stats models library was used [26]. Graphs were generated programmatically using the seaborn library [27]. Secondly, we quantified the overlap of the top 20 responders among the fresh and cryopreserved cells, as the number of substances, which in both sets results in the 20 lowest GI50 concentrations, thereby calculating relative repeatability.

## 5. Conclusions

We established a robotic-assisted throughput in vitro drug screening technology suitable to perform assays on cells that require feeder layer support for their optimal growth. Although some variations in the absolute drug effects between the tested time points occur, the relative efficacy of drugs that most strongly reduce the growth of the tested hiPSC line (=top 20) remains stable in terms of their effects on cells before and after cryopreservation, when interrogating the total variances that occur in the data obtained from all the >160 tested drugs. This also applies to cells that are genetically engineered in order to overexpress oncogenes. Our project indicates that hiPSCs support the development of reliable in vitro drug-testing platforms aiming to identify biomarker-related resistances. 

## Figures and Tables

**Table 1:** The list of stable and unstable drugs for IPS11-wild type.

Stable (Drugs That Show up in Top 20 List before and after Freezing)			Unstable (Drugs That Show up as Top 20 Performer Only before Freezing Whereas after That They Fall behind the Threshold)	
Drug Name	Rank before Freezing	Rank after Freezing	Drug	Rank before Freezing
Cabazitaxel	3	3	Ixabepilone	1
Docetaxel	4	12	Decitabine	2
Topotecan hydrochloride	5	5	Gemcitabine	6
Erlotinib	7	7	Nintedanib Ethanesulfonate Salt	9
Homoharringtonine	8	11	Mitoxantrone hydrochloride	12
Podophyllotoxin	10	8	Vinblastine sulfate	13
Imiquimod	11	10	Paclitaxel	15
Acalabrutinib	14	15	Romidepsin	16
			Nintedanib	17
			Carfilzomib	18
			Lenalidomide	19
			Sumatriptan succinate	20

**Table 2:** The list of stable and unstable drugs for IPS11-pSin-TP53.

Stable (Drugs That Show up in Top 20 List before and after Freezing)			Unstable (Drugs That Show up as Top 20 Performer Only before Freezing Whereas after That They Fall behind the Threshold)	
Drug Name	Rank before Freezing	Rank after Freezing	Drug	Rank before Freezing
Ixabepilone	1	4	Ibrutinib	2
Cabazitaxel	3	17	Docetaxel	4
Podophyllotoxin	5	10	Vinblastine sulfate	6
Homoharringtonine	7	13	Decitabine	12
Doxorubicin hydrochloride	8	16	Nintedanib	13
Paclitaxel	9	1	Erlotinib	18
Panobinostat	10	3	Topotecan hydrochloride	19
Bortezomib	11	5		
Carfilzomib	14	12		
Nintedanib Ethanesulfonate Salt	15	6		
Romidepsin	16	8		
Acalabrutinib	17	19		
Mitoxantrone hydrochloride	20	9		

**Table 3:** The list of stable and unstable drugs for IPS11-pSin-cMYC.

Stable (Drugs That Show up in Top 20 List before and after Freezing)			(Drugs That Show up as Top 20 Performer Only before Freezing Whereas after That They Fall behind the Threshold)	
Drug Name	Rank before Freezing	Rank after Freezing	Drug	Rank before Freezing
Doxorubicin hydrochloride	3	3	Nintedanib Ethanesulfonate Salt	1
Ixabepilone	4	2	Cabazitaxel	2
Mitoxantrone hydrochloride	5	8	Nintedanib	6
Homoharringtonine	7	14	Acalabrutinib	15
Podophyllotoxin	8	12	Paclitaxel	16
Topotecan hydrochloride	9	13	Erlotinib	18
Vinblastine sulfate	10	7	Palbociclib Isethionate	19
Docetaxel	11	11	Dasatinib	20
Panobinostat	12	20		
Bortezomib	13	18		
Carfilzomib	14	5		
Romidepsin	17	16		

## Data Availability

Data is contained within the article.
